# Epidemiologic, Clinical, and Laboratory Findings of the COVID-19 in the current pandemic

**DOI:** 10.21203/rs.3.rs-28367/v1

**Published:** 2020-05-28

**Authors:** Yewei Xie, Zaisheng Wang, Huipeng Liao, Gifty Marley, Dan Wu, Weiming Tang

**Affiliations:** University of North Carolina at Chapel Hill-China Project; University of North Carolina at Chapel Hill-China Project; University of North Carolina at Chapel Hill-China Project; Nanjing Medical University; London School of Hygiene and Tropical Medicine

**Keywords:** COVID-19, Epidemiology, Nature history, Clinical spectrum

## Abstract

**Background:**

The COVID-19 caused the pandemic affected the world deeply, with more than 3,000,000 people infected and nearly 200,000 deaths. This article aimed to summarize the epidemiologic traits, clinical spectrum, CT results and laboratory findings of COVID-19 pandemic.

**Methods:**

We scoped for relevant literatures published during 1st Dec 2019 to 23rd Apr 2020 based on four databases by using English and Chinese. The evidence was synthesized narratively.

**Results:**

The COVID-19 pandemic was found to have a higher transmission rate compared to SARS and MERS, and involved 4 stages of evolution. The basic reproduction number (R0) is 3.32 (95% CI:3.24–3.39) and the incubation period was 5.24 days (95% CI:3.97–6.50, 5 studies) on average, and the average time for symptoms onset varied by countries. Common clinical spectrums identified included fever (38.1–39.0°C), cough and fatigue, with Acute Respiratory Distress Syndrome (ARDS) being the most common complication reported. Body temperatures above 39.0 °C, dyspnea, and anorexia were more common symptoms in severe patients. Aged over 60 years old, having co-morbidities, and developing complications were the commonest high-risk factors associated with severe conditions. Leucopenia and lymphopenia were the most common signs of infection while liver and kidney damage were rare but may cause bad outcomes for patients. The bilateral, multifocal Ground-Glass Opacification (GGO) on peripheral, and the consolidative pulmonary opacity were the most frequent CT results and the tendency of mortality rates differed by region.

**Conclusions:**

We provided a bird’s-eye view of the COVID-19 during the current pandemic, which will help better understanding the key traits of the disease. The findings could be used for disease’s future research, control and prevention.

## Background

The emergence of COVID-19 has made it the first infectious disease pandemic in the 21 st century. As of April 23rd, a total of 2,544,792 COVID-19 cases has been reported and confirmed with 175,694 deaths globally. While more than 30 countries had issued the highest level of response, the SARS-CoV-2 (pathogen of COVID-19) continues to spread in different regions around the world [W1]. However, the key information on the virus epidemiology, clinical spectrum, and on the pathogen were delayed in response during the early outbreaks in many countries. To fill the research gaps mentioned above, this article systematically summarizes global findings on the natural history, clinical spectrum, transmission patterns, laboratory findings, CT results, and risk factors of the COVID-19.

## Methods

### Information sources, search and study selection

We searched for publications in epidemiology and clinic domains of the COVID-19 broadly as it was a novel, rapidly extended, infectious disease. Both the English and Chinese were used in searching as the COVID-19 was firstly reported in Wuhan, most of the real-time study were in Chinese. Then, we searched the following databases: CHKD v3.1 of the CNKI [in Chinese], PubMed, Web of Science, and medRxiv&bioRxiv by using search terms ‘COVID-19, SARS2, SARS-CoV-2, 2019 nCoV’ (Appendix 1). The publication date was restricted from 1 st Dec 2019 to 23rd Apr 2020. Only the full-text available human studies were eligible for selection. Real-time data were obtained from health departments of multiple countries, global NGOs, and reputable media sources.

### Data collection process

**Figure 1:**
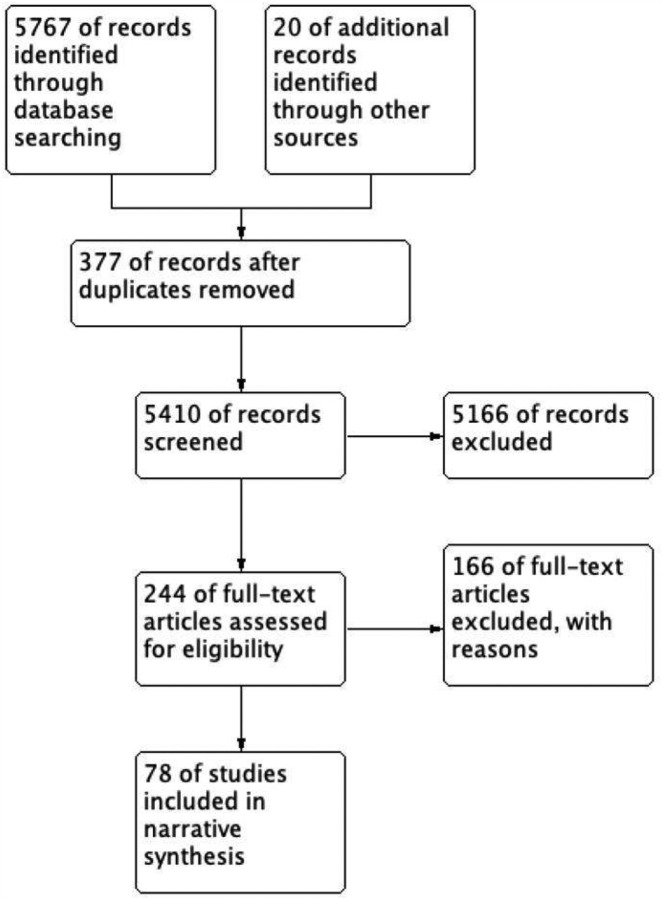
PRISM flow diagram

Three review authors (HL, YX, and ZW) reviewed the titles and abstracts of all searched records in the first screening. The full-text articles were reviewed for further screening. The full-text records would submit to the reviewer (DW and WT) to consult and make final decisions when there were disagreements. The excluded criteria were listed in ‘Characteristics of excluded studies table (Appendix 2)’, and the included studies were listed in the ‘Included studies table (Appendix 3)’. Besides, a PRISMA diagram was conducted to illustrate the entire flows of this literature review (Fig. 1).

Four authors (HL, WT, YX, and ZW) extracted data from the included studies independently. When there were disagreements, the data were handed to the full members of the review group for further discussion and resolved. At last, the confirmed data will be entered into Review Manager 5 (RevMan 5) and double-checked by all review authors in the team.

The Cochrane Handbook for Systematic Reviews of Interventions suggested review authors collect missing data from investigators the missing data. It was because the missing data could lead to the bias during analyzing. However, using the imputation method to tackle the missing data problem could not reduce bias [W2]. Therefore, we only analyze data available to us if we could not collect the missing data from the investigators.

### Dealing with missing data

The Cochrane Handbook for Systematic Reviews of Interventions suggested review authors collect missing data from investigators the missing data. It was because the missing data could lead to the bias during analyzing. However, using the imputation method to tackle the missing data problem could not reduce bias [W2]. Therefore, we only analyze data available to us if we could not collect the missing data from the investigators.

### Data synthesis, and assessment of heterogeneity and reporting biases

We reviewed all kinds of researches about the COVID-19 outbreak systematically from the beginning until the latest date before we submitted this paper. Because the objective of this study aimed to get a bird view of the COVID-19 outbreak, and updated the clinical spectrum and natural history of the COVID-19, we synthesis data narratively. Thus, to assess the heterogeneity and reporting bias of the included studies.

## Results

We collected 5767 records from 4 databases (CNKD, Pubmed, Web of Science, and medVix&BioVix), and 20 records from other resources. 5410 records remained after removing 377 duplications. After two batches of screening, 78 records were included in this review.

### Epidemiology

#### Demographic characteristics

In a 55,924 COVID-19 patients study based in China, the majority of patients aged 30–69 (77.8%) with only 2.4% of the patients being 18 years and below. The median age of the patients was 51 (ranged 2 days-100 years old) [W3]. Similarly, in the United States, more than half of patients aged between 20–64 years (65%), and patients under 19 only accounted for 5% of all patients. Older aged patients were more prone to getting infected compared to the young [1]. The US’s data also indicated that patients younger than 19 had milder COVID-19 illness, with almost no hospitalizations or deaths reported [W4]. Based on a worldwide data, the elderly (aged over 60) were at a high risk of developing into severe disease or death [W3-W5, 2]. About the gender ratio, the male to female ratio of confirmed cases was 1.06:1.00 in China [3]. However, in South Korea and Iceland, the male population had a higher incidence rate than the female population [1, 2]. Males had double times the secondary attack rate than females [4].

#### Transmission stages

Globally, the transmission of the COVID-19 can be categorized into 4 temporal stages. The first stage: people with exposure histories to Huanan Seafood Market (HSM) got infected [5]. Forty-one patients were found to be having SARS-like symptoms in December 2019, and the HSM was believed to be one of the origins of the virus. However, 13 of the 41 patients reported no prior exposure to the HSM thus indicating that the origin of the virus needed further investigation [6]. The second temporal stage is the transition from community transmissions to the outbreak in Wuhan [5]. The virus was mainly spread to multiple communities directly and indirectly by people with HSM exposure histories. The interpersonal transmissions and clustered transmissions formed community transmissions [5]. An early study showed that the proportion of patients with HSM exposure histories decreased from 55–8.6% within 22 days, indicating people who did not have exposure histories to the HSM became infected [7, 8]. The third stage: the epidemic in China. At this stage, transmissions began to expand to communities outside Wuhan and the Hubei province as a whole [5]. On 26th Jan 2020, a study involving 62 COVID-19 patients outside Wuhan found that all the patients had been exposed to Wuhan, which demonstrated an established local transmission outside Wuhan [9]. The fourth temporal stage is the global pandemic. On 13th Jan 2020, the first case outside China was reported in Thailand [W1]. On 30th Jan 2020, the WHO declared a Public Health Emergency of International Concern (PHEIC) [W1]. It subsequently took about 51 days for transmission to escalate from the first reported case to the 10,000th reported case outside China. Globally, it took 16 days for the number of reported cases to increase from 10,000th cases to 100,000th cases, 21 days from 100,000th cases to 500,000th cases, only 6 days from 500,000th cases to 1,000,000th cases and 13 days from 1,000,000th cases to 2,000,000th cases [W1].

#### Transmission Routes

The main transmission form of this virus was by human-to-human spread, since only 1.18% patients among 1099 confirmed patients had history of direct contact with wild animals [10]. The vital transmission routes were through respiratory droplets and contact transmissions. There remains the possibility of aerosol transmission when exposed to high concentrations of aerosols for a long time in a relatively closed environment [W6]. Fecal-oral transmission and the mother-to-child transmission were also possible but lacked direct evidence until now [11, 12]. Other suspected routes of transmission still needed to be clarified.

#### Transmission Patterns

The transmission patterns of COVID-19 included community transmission, nosocomial transmission, household transmission, and transmission in closed environments.

Firstly, the community transmission was considered to be an important pattern in COVID-19 spread [W3]. In the Netherlands, community transmissions were found in the Noord-Brabant regions [W7, W8]. In North America, community transmissions were reported in Winnipeg, Canada, and Eastern Idaho, United States [W9, W10]. Secondly, the potential risk of transmission among medical personnel and through medical facilities was deemed high and thus should be paid extreme attention.

Transmissions between patients and health workers were in higher proportion during the SARS outbreak, while transmission through medical facilities was higher proportion during the MERS outbreak [13]. In Wuhan, the proportion of severely infected medical workers was higher than the national average [3]. In Italy, 2,629 health workers were reported infected with the COVID-19 before 18th March and accounted for 8.3% of the total number of cases nationwide. The number however increased to 8,358 by 30th March and represented 9% of the country’s total number of cases [W11, W12]. In Spain, the number of diagnosed cases among medical workers increased to 5,969 within 6 days and more than 12% of the country’s confirmed cases remained among medical workers until March 30th [W12]. Update from another source reported an increase in the number of cases from 12–14% among Spain healthcare workers by 31st March and this was attributed to lack of medical supplies, such as masks and gowns. Other reasons accounting for these high infection rates among medical personnel varied according to different country’s circumstances. An Italy study pointed out hospitals as a potential hotspot for infection. Facilities and medical personnel turned into untested vectors and patients [W13, W14]. In the US for example, the reasons that turned hospitals into infection hotspots included the overload of COVID-19 patients and inappropriate management against the pandemic in hospitals [W15]. Similar to the US, 200 medical workers got infected in a county hospital in Romania due to inadequate hospital management. In Egypt, a serious wave of emigration by physicians for years led to patient overload for remaining medical workers and placed them at higher risk of infection through continuous exposure. The emigration wave was purportedly caused by low salary, undesirable working conditions, lack of legal protection, and shortage of medical supplies and equipment [W16].

Thirdly, household transmission contributed to cluster infections and was the major transmission pattern observed in China. For instance, among 1836 reported cases in Guangdong and Sichuan Provinces, most cluster infections occurred in families (78%−85%) [W3]. The WHO in this regard issued a statement that household transmission highly occurred among medical workers’ families than health facility infection in China. Household transmission was also a significant pattern observed in South Korea and the US [W1, W15]. The European Centre for Disease Prevention and Control (ECDC) had provided guidance for the control of household transmission in European countries [W3, W17]. What made household transmission worse was that some groups (age <18 and > 65) had high risk got infection within households than the general population [W18]. So, children and elderly living with medical workers at a higher risk of getting than other populations.

Fourthly, transmissions in a closed environment besides the home should also be of a keen focus on the prevention and control of this outbreak. A Japanese health department reported that a closed environment could promote super-spreading events because the transmission of the SARS-CoV-2 in a closed environment was the same as large-scale transmission, such as the ski chalet-cluster infection in France and the church-hospital infection clusters in South Korea [W19]. For example, outbreaks of the COVID-19 were observed in multiple prisons in China, the UK, and the US [W20, W21, 3]. Cluster infections also happened on cruise ships, such as the Diamond Princess, Grand Princess, Golden Princess, Ruby Princess, Phoenix Reisen, MS Westerdam, and Punta Arenas [W22]. Further studies are however required to identify and assess other potential transmission patterns for further prevention, especially since some cases were asymptomatic [15, 16]. In addition, patients who were considered cured and no longer needed quarantine still tested RT-PCR positive after 5 to 13 days [17].

#### Nature history

We systematically pooled data on the incubation period and the reproduction numbers for analysis. The pooled data suggested that the mean incubation period was 5.24 days (95% CI:3.97–6.50, 5 studies), and ranged from 3–7.4 days [W23-W27]. However, the incubation period in some special cases could be as long as 24 days [10]. The pooled results also illustrated that the basic reproduction number (R0) of SARSCoV-2 was 3.32 (95% CI:3.24–3.39, 14 studies) and varied between 0.6–6.47 [W24-W35, 15]. The results suggested that the transmission ability of SARS-CoV-2 was stronger than SARS (3) and MERS (≤ 1) [W36, W37]. Moreover, the median time from the first symptom to first hospital admission was 7 days with the median duration from illness development to severe symptoms development being: 5–8 days for dyspnea, 8–9 days for ARDS, 10.5 days for mechanical ventilation and ICU admission [6, 18]. For COVID-19 related deaths, the duration from the onset of symptoms to death averaged 9 days in China 5 and in Italy (median) [W5], and 10 days in South Korea (median) [2].

#### Mortality

**Figure 2:**
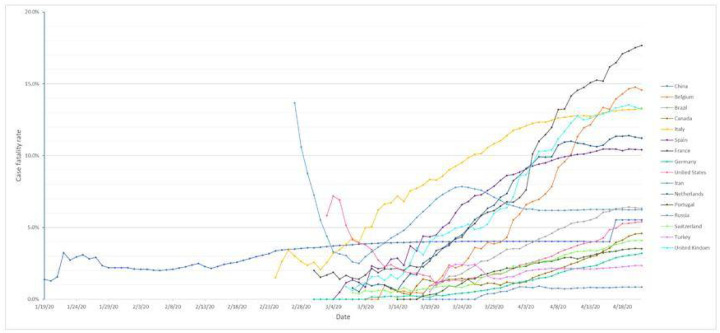
Case fatality rate of countries reported over 20,000 cases, 2020* *Data was collected until 21 April 2020. The CFR of a country was not included on those dates when the country reported less than 100 cases, with the consideration that the CFR may not be reliable if the size of infected population was small

By 21 st Apr, 16 nations had reported over 20,000 COVID-19 cases in each of the countries, together contributed to 84.6% of the confirmed cases and 92.4% of death in the world [W38]. The global Case Fatality Rate (CFR) was 7.0% on 21st Apr. However, it was apparently different by country. Among the 16 countries, 6 had over 10% CFR with an apparently increasing trend after reported the over-100 cases (Fig. 2). These countries were France (17.7%), Belgium (14.6%), Italy (13.3%), United Kingdom (13.2%), Netherlands (11.2%), and Spain (10.4%), which were all from the European region. Among the other 10 countries, the CFR of Brazil, Canada, Switzerland, Portugal, and Germany slowly increased to 6.3%, 4.6%, 4.1 %, 3.5%, and 3.2%, respectively; the CFR of China, Russia, and Turkey was relatively stable at 5.5%, 0.9%, and 2.4%, respectively. The CFR of Iran and the United States largely fluctuated. The CFR of Iran firstly declined to 2.5% on 8th Mar., then increased to 7.9% on 24th Mar., and slowly decreased again and became stable at 6.2% on 21 st Apr. The CFR of the United States decreased to 1.1 % on 20th Mar. and then slowly increased to 5.4% on 21st Apr. As the pandemic outbreak continued, more surveillance is needed for the CFR of COVID-19.

#### Common symptoms and Complications

Fever (83.0%−90.9%) and cough (57.6%−70.8%) were the most common symptoms of the COVID-19 in China. Other common symptoms included: fatigue (29.4%−41.0%), dyspnoea (14.5%−45.6%), sputum production (28.7%−32.6%), sore throat (14.0%−14.1%), etc. Nausea and vomiting (5.4%−8.9%), and diarrhea (4.7%−6.1%) were atypical symptoms [19–22]. Besides, a study pointed out that most patients (90%) had more than one symptom [18]. Additionally, there were 20.9% of patients without viral pneumonia symptoms [10], which was opposite to previous studies [6, 18, 23].

However, in Italy’s report, the most commonly observed symptoms were fever and dyspnea whiles cough, diarrhea and hemoptysis were less common. Overall, 6.4% of patients did not present any symptoms during hospital admissions [W5]. In Europe, one study found olfactory and gustatory disorders were common symptoms, which were totally different from China’s findings [24]. In the US, the gastrointestinal symptoms were shown more frequently than in China [25].

**Figure 3:**
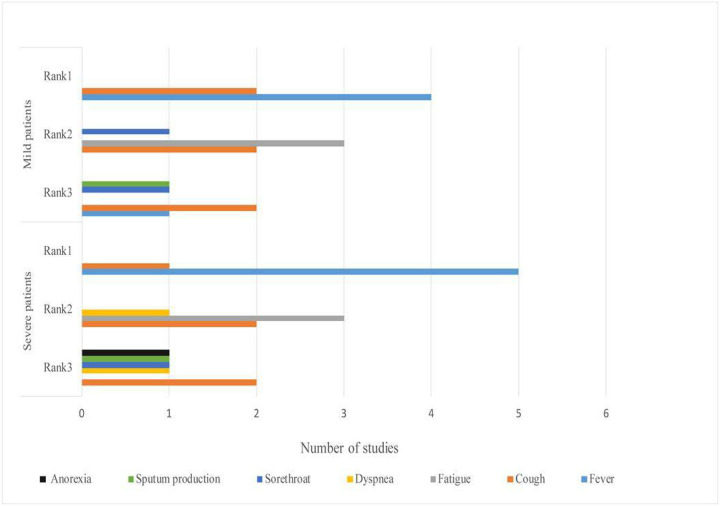
Comparison of top 3 symptoms among mild and severe patients with COVID-19, 2020* *The X-axis means the number of symptoms reported by how many studies. The Y-axis means symptoms’ ranking in mild and severe patients

The top 3 common symptoms among mild and severe patients are summarized and displayed in a figure (Fig. 3) [6, 10, 18, 26–30]. Fever was found to be the most common symptom in all patients. In a study, 43.8% of patients had fever initially and the proportion increased to 87.9% following hospitalization [10]. The body temperatures of 44%−47.1 % of patients ranged between 38.1–39.0°C. The higher body temperatures (above 39.0°C) as well as dyspnea and anorexia were more frequent among patients in severe conditions only [6, 10, 29]. Cough and fatigue on the other hand were more widely reported among mild and severe patients. What’s more, another study reported that dyspnea (76%) was the most common symptom among severe patients in the United States [31]. The proportion of patients who needed ICU care varied based on the local pandemic circumstances. For example, the WHO speculated that around 13.8% of patients were in severe conditions in China [W3]. However, 23%−32% of patients needed ICU care in Wuhan [6, 18, 23].

Currently documented COVID-19 related complications include ARDS, RNA-anemia, arrhythmia, shock, acute cardiac injury, acute respiratory injury, acute renal injury, and secondary infection, etc. The ARDS was the most common complication, with incidence rate ranging from 8.9–32.8% in China [6, 18–23] and 96.4% in Italy [W5]. The progress of some patients with ARDS to septic shock was fast and quickly evolved into multiple organ failure finally [23]. Most ICU patients had a higher risk of developing ARDS and having complications [6, 18].

### Laboratory findings and CT Scans

#### Laboratory findings

Among COVID – 19 patients, a decrease in leukocytes such as eosinophil and lymphocyte were commonly reported. This might be because the cytokine storm caused by the novel virus changes the peripheral of white blood cells and immune cells [6, 8–10, 18, 23]. Severe lymphopenia was also common among the dead patients [6, 30]. Myocardial zymogram abnormality was found in many patients. For instance, 76% of patients had an increase in lactate dehydrogenase, while 13% of patients had increases in creatine kinase [23]. The level of C-reactive protein was important to evaluate the infection [10]. Most patients were found to have a higher level of C- reactive protein (86%) and serum ferritin (63%) compared to the normal range [23]. The biomarkers related to liver and renal damage were found to be abnormal among COVID-19 patients. The abnormality of liver-related biomarkers was not widespread but yet still common in severe cases [6, 9,10, 32]. Besides, although only 7% of patients showed renal biomarker abnormalities, renal damage might contribute to the final multi-organ failure and death outcome [33, 34].

The ICU patients showed higher levels of white blood cells, neutrophil counts, D-dimer, creatine kinase, and creatine with longer prothrombin times [6, 10, 18]. Compared to patients who survived, the patients who died had higher levels of D-dimer, high-sensitivity cardiac troponin I, serum ferritin, lactate dehydrogenase, IL-6, blood urea, creatinine, white blood cell counts and neutrophil counts. Severe lymphopenia was also common among dead patients [6, 30].

#### Computed Tomography Scan (CT Scan) features

Most patients had GGO and the bilateral lung involvement [6, 23, 35–37]. One study found that bilateral lung involvement was more frequently shown in the intermediate course and late course, compared to the earlier clinical course [38]. The clinical course could be divided into four stages based on CT scan findings [37]. In the first stage (Pre-symptom), GGO, unilateral and multifocal were observed among most patients in this stage [37, 38]. In the second stage (symptoms ≤1 week), lesions soon developed into bilateral and diffused except for GGO. This stage was considered a period from transition to consolidation. A mixed pattern of transition and consolidation develops during this stage. In the third stage (symptoms 1–2 weeks), the GGO was still common and the consolidation pattern showed. Findings indicated an interstitial change, which was considered as the development of fibrosis. In the fourth stage (symptom 2–3 weeks), consolidation and mixed patterns were more common, and the GGO started to shrink [37], the consolidation was gradually absorbed among patients who recovered at last [39].

Among ICU patients, the bilateral multiple lobular and subsegmental areas of consolidation were considered typical findings [6]. Patients in severe condition showed diffuse lesions, with density increasing in both lungs. CT scans showed ‘white lung’ appearances, indicating the serious influence the infection has on patients’ lung functions [40].

#### Risk factors

Being old (≥ 65 years old), having comorbidities (e.g. hypertension, diabetes, cardiovascular and cerebrovascular diseases, etc.), and developing complications were three vital risk factors for patients to develop severe conditions [18, 26, 27]. Findings from multiple studies have shown that patients who are more than 65 years of age, and with comorbidities such as diabetes and heart diseases had a high mortality rate [26, 30, 41–43]. Late hospitalization and bacterial infections were also considered high risk factors for disease progression [23, 27, 42]. Smoking history could be a potential risk factor for developing severe conditions [23, 27]. People with underlying disorders were considered to be at a high risk of getting infected [W3].

## Discussion

Our review identified several research gaps. Firstly, large amounts of data for countries outside of China were missing from this review. This is because the first case of COVID-19 was found in China and lasted for three months, while other countries were still at the early stages of the epidemic, except for South Korea [W38]. Besides, published studies of coronavirus in China grew rapidly since the SARS outbreak in 2002, maintaining a top 2 position in the world [44]. Secondly, although mother-to-child transmission was conceded to be a possible transmission route [W39], no direct evidence has proved this and further investigation is needed [3, 45]. Besides, there exist unknown transmission patterns that need to be identified in future studies. Third, a ‘super-spreader’ was defined as infected individuals who made infected numerous others during the SARS outbreak. For example, a nephrotic hospitalized patient was defined as a ‘super-spreader’ during the SARS in China, causing 22 people infected. 19 in 22 patients were the medical workers contacted with the ‘super-spreader’. The incident rare among the medical workers was 59.38% (19/32) in the nephrotic department [46]. In the COVID-19, ‘super-spreaders’ were found in multiple places worldwide. A Saudi Arabic study issued the ‘super-spreader’ to ‘superspreading event’. The study mentioned that ‘super-spreader’ might cause unexpected transmission during the pilgrimage [47], when huge number of people gathering with a high population density. Reasons causing the super- spreading event might include: immune suppression, increased disease severity and viral load, asymptomatic individuals, and extensive social interactions [48]. However, the characteristics and features of how an individual became a super-spreader were not clear [W40]. Summarizing the features of the ‘super spreader’ concept, as well as their characteristics and role in transmissions, are needed in future disease control [49]. Fourth, it has been reported that some cured patients COVID-19 retested positive by PCR after being discharged and quarantined at home in multiple places [W41, 17]. The reason for this phenomenon is still unclear and hence further investigations are required for future pandemic control [W42].

There existed some limitations in this review. Firstly, this review base on the English and Chinese resource. As the COVID-19 transform to global pandemic from regional outbreak, comprehensive collection of the related information worldwide is needed. Secondly, the clinical spectrum based on general population, while subgroup analyzes may help to figure more entire picture of the COVID-19. For instance, Kawasaki disease were found in children in the UK and Europe countries [W43]. However, other places did not report the gathering Kawasaki disease cases as the UK and Europe.

## Conclusions

By collecting and analyzing published COVID-19 articles, we found the average incubation period of COVID-19 was 5.24 days, the R0 was 3.32, indicated a stronger transmission ability than SARS and MERS. The common symptoms had a little difference in Europe and Asia. Body temperatures above 39.0 °C, dyspnea, and anorexia were more common symptoms in severe patients. Age over 60 years old, having co-morbidities, and developing complications were high-risk factors developed into severe conditions or death. A decrease in leukocytes was common, the abnormal lab biomarkers of cardiac, liver, and renal were found. Observed from the CT findings, GGO and the bilateral lung involvement were common, the longer the time after symptoms onset, the more consolidated lung lesions were found.

## Supplementary Material

Supplement

## Abbreviations

ARDS: Acute Respiratory Distress Syndrome
CFR: Case Fatality Rate
COVID-19: Coronavirus disease 2019
CT: Computed Tomography Scan
ECDC: The European Centre for Disease Prevention and Control
GGO: Ground-Glass Opacification
ICU: Intensive care unit
MERS: Middle East respiratory syndrome-related coronavirus
PCR: Polymerase chain reaction
R0: Basic reproduction number
SARS: Severe acute respiratory syndrome
SARS-CoV-2: Severe acute respiratory syndrome coronavirus 2
WHO: World Health Organization
