# Supplementary material for: Epidemiologic, Clinical, and Laboratory Findings of the COVID-19 in the current pandemic
Source: Res Sq. 2020 May 28:rs.3.rs-28367. Preprint. [Version 1] doi: 10.21203/rs.3.rs-28367/v1 (PMC7336699; doi:10.21203/rs.3.rs-28367/v1)
Supplement: Supplementary file 1 [file AppendixMay5.docx]

**Appendix**

**Appendix 1 Searching Terms**

PubMed

| **Search** | **Query** | **Items found** |
| --- | --- | --- |
| [#6](https://pubmed.m7h.net/pubmed/advanced) | Search #4 or #5 Filters: Publication date from 2019/12/01 to 2020/04/23 | [2788](https://pubmed.m7h.net/pubmed/?cmd=HistorySearch&querykey=11) |
| [#5](https://pubmed.m7h.net/pubmed/advanced) | Search #1 and #3 Filters: Publication date from 2019/12/01 to 2020/04/23 | [2075](https://pubmed.m7h.net/pubmed/?cmd=HistorySearch&querykey=9) |
| [#4](https://pubmed.m7h.net/pubmed/advanced) | Search #1 and #2 Filters: Publication date from 2019/12/01 to 2020/04/23 | [1195](https://pubmed.m7h.net/pubmed/?cmd=HistorySearch&querykey=8) |
| [#3](https://pubmed.m7h.net/pubmed/advanced) | Search clinic* Filters: Publication date from 2019/12/01 to 2020/04/23 | [183057](https://pubmed.m7h.net/pubmed/?cmd=HistorySearch&querykey=7) |
| [#2](https://pubmed.m7h.net/pubmed/advanced) | Search epidemiolog* Filters: Publication date from 2019/12/01 to 2020/04/23 | [38051](https://pubmed.m7h.net/pubmed/?cmd=HistorySearch&querykey=6) |
| [#1](https://pubmed.m7h.net/pubmed/advanced) | Search (((COVID-19[Title/Abstract]) OR SARS2[Title/Abstract]) OR SARS-CoV-2[Title/Abstract]) OR 2019 nCo[Title/Abstract] Filters: Publication date from 2019/12/01 to 2020/04/23 | [6427](https://pubmed.m7h.net/pubmed/?cmd=HistorySearch&querykey=3) |

CHKD

| 主题='新型冠状病毒' or 主题='新型冠状病毒肺炎 ' or 主题='新冠肺炎' and 主题='流行病学' or 主题='临床' 不包含 主题='中医' or 主题='中药 ' or 主题='针灸 ' or 主题='药理学 ' or 主题='心理' and 发表时间 between ('2019-12-01','2020-04-23') (精确匹配) | 1,442 |
| --- | --- |

medRxiv and bioRxiv

| ((((COVID-19) OR SARS2) OR SARS-CoV-2) OR 2019 nCo) and (clinical or epidemiology))" and posted between "01 Dec, 2019 and 23 Apr, 2020 | 1004 |
| --- | --- |

Web of Science

| ALL FIELDS: (COVID-19 or 2019-nCov or SARS-Cov-2 or SARS2) AND ALL FIELDS: (clinical or epidemiology) Timespan: 2019-2020. | 553 |
| --- | --- |

**Appendix 2 Characteristics of excluded studies**

| **Table 1: Characteristics of excluded studies** |  |  |
| --- | --- | --- |
| 1 | de Freitas E Silva R 2020 | Insufficient clinical data. |
| 2 | Fang, Y 2020 | Insufficient clinical data. |
| 3 | Kurtkoti R 2020 | Insufficient epidemological data. |
| 4 | Lal A 2020 | Insufficient clinical data. |
| 5 | Maddaloni E 2020 | Insufficient clinical data. |
| 6 | Martinez-Alvarez M 2020 | Insufficient epidemological data. |
| 7 | Nouri-Vaskeh M 2020 | Insufficient epidemological data. |
| 8 | Okba NMA 2020 | Virological study. |
| 9 | Payne C 2020 | Insufficient epidemological data. |
| 10 | Qanadli SD 2020 | Virological study. |
| 11 | Rubin R 2020 | Opinion but not the epidemological study. |
| 12 | Shah J 2020 | Insufficient epidemological data. |
| 13 | Szymanek-Pasternak A 2020 | Insufficient clinical data. |
| 14 | Torres I 2020 | Insufficient epidemological data. |
| 15 | Wang, X 2020 | Insufficient clinical data. |
| 16 | Wu, P 2020 | Insufficient epidemological data. |

**Appendix 3 Included Studies**

| **Table 2 Included Studies** |  |  |
| --- | --- | --- |
| 1 | Gudbjartsson et al. 2020 | Spread of SARS-CoV-2 in the Icelandic Population |
| 2 | Choe 2020 | Coronavirus disease-19: The First 7,755 Cases in the Republic of Korea. |
| 3 | Epidemiology Working Group for NCIP Epidemic Response 2020 | The epidemiological characteristics of an outbreak of 2019 novel coronavirus diseases (COVID-19) in China. Chinese Journal of Epidemiology. |
| 4 | COVID-19 National Emergency Response Center 2020 | Coronavirus disease-19: Summary of 2,370 contact investigations of the first 30 cases in the Republic of Korea. |
| 5 | Special Expert Group for Control of the Epidemic of Novel Coronavirus Pneumonia of the Chinese Preventive Medicine Association 2020 | An update on the epidemiological characteristics of novel coronavirus pneumonia (COVID-19). |
| 6 | Chen et al. 2020 | Clinical characteristics and intrauterine vertical transmission potential of COVID-19 infection in nine pregnant women: a retrospective review of medical records. |
| 7 | Li et al. 2020 | Early transmission dynamics in Wuhan, China, of novel coronavirus–infected pneumonia. |
| 8 | Zhang et al. 2020 | Clinical characteristics of 140 patients infected with SARS‐CoV‐2 in Wuhan, China. |
| 9 | Xu et al. 2020 | Clinical findings in a group of patients infected with the 2019 novel coronavirus (SARS-Cov-2) outside of Wuhan, China: retrospective case series. |
| 10 | Guan et al. 2020 | Clinical characteristics of 2019 novel coronavirus infection in China. |
| 11 | Yeo et al. 2020 | Enteric involvement of coronaviruses: is faecal–oral transmission of SARS-CoV-2 possible? |
| 12 | Chen et al. 2020 | Clinical characteristics and intrauterine vertical transmission potential of COVID-19 infection in nine pregnant women: a retrospective review of medical records. |
| 13 | Chowell et al. 2020 | Transmission characteristics of MERS and SARS in the healthcare setting: a comparative study. |
| 14 | Burke et al. 2020 | Active monitoring of persons exposed to patients with confirmed COVID-19—United States, January–February 2020. |
| 15 | Pan et al. 2020 | Asymptomatic cases in a family cluster with SARS-CoV-2 infection. |
| 16 | Bai et al. 2020 | Presumed asymptomatic carrier transmission of COVID-19. |
| 17 | Lan et al. 2020 | Positive RT-PCR test results in patients recovered from COVID-19. |
| 18 | Wang et al. 2020 | Clinical Characteristics of 138 Hospitalized Patients With 2019 Novel Coronavirus-Infected Pneumonia in Wuhan, China. |
| 19 | Sun et al. 2020 | Clinical Characteristics of 5732 Patients with 2019-nCoV Infection. |
| 20 | Rodriguez-Morales et al. 2020 | Clinical, laboratory and imaging features of COVID-19: A systematic review and meta-analysis. |
| 21 | Fang et al. 2020 | Clinical Characteristics of 2019 Coronavirus Pneumonia (COVID-19): An Updated Systematic Review. |
| 22 | Lin, Wen, and Chen 2020 | Epidemiological and clinical characteristics of SARS-CoV-2 and SARS-CoV: a system review. |
| 23 | Chen et al. 2020 | Epidemiological and clinical characteristics of 99 cases of 2019 novel coronavirus pneumonia in Wuhan, China: a descriptive study. |
| 24 | Lechien et al. 2020 | Olfactory and gustatory dysfunctions as a clinical presentation of mild-to-moderate forms of the coronavirus disease (COVID-19): a multicenter European study. |
| 25 | Goyal et al. 2020 | Clinical Characteristics of Covid-19 in New York City. |
| 26 | Yang et al. 2020 | Clinical course and outcomes of critically ill patients with SARS-CoV-2 pneumonia in Wuhan, China: a single-centered, retrospective, observational study. |
| 27 | XIANG et al. 2020 | Analysis of clinical characteristics of 49 patients with Novel Coronavirus Pneumonia in Jiangxi province. |
| 28 | Young et al. 2020 | Epidemiologic features and clinical course of patients infected with SARS-CoV-2 in Singapore. |
| 29 | Zhang et al. 2020 | Clinical features and outcomes of 221 patients with COVID-19 in Wuhan, China. |
| 30 | Zhou et al. 2020 | Clinical course and risk factors for mortality of adult inpatients with COVID-19 in Wuhan, China: a retrospective cohort study. |
| 31 | Arentz et al. 2020 | Characteristics and Outcomes of 21 Critically Ill Patients With COVID-19 in Washington State. |
| 32 | Liu et al. 2020 | Preliminary study of the relationship between novel coronavirus pneumonia and liver function damage: a multicenter study. |
| 33 | Wang et al. 2020 | Caution on Kidney Dysfunctions of 2019-nCoV Patients. |
| 34 | Cheng et al. 2020 | Kidney impairment is associated with in-hospital death of COVID-19 patients. |
| 35 | Song et al. 2020 | Emerging Coronavirus 2019-nCoV Pneumonia. |
| 36 | Xu et al. 2020 | Imaging and clinical features of patients with 2019 novel coronavirus SARS-CoV-2. |
| 37 | Shi et al. 2020 | Radiological findings from 81 patients with COVID-19 pneumonia in Wuhan, China: a descriptive study. |
| 38 | Bernheim et al. 2020 | Chest CT Findings in Coronavirus Disease-19 (COVID-19): Relationship to Duration of Infection. |
| 39 | Pan et al. 2020 | Time Course of Lung Changes On Chest CT During Recovery From 2019 Novel Coronavirus (COVID-19) Pneumonia. |
| 40 | Pan et al. 2020 | Initial CT findings and temporal changes in patients with the novel coronavirus pneumonia (2019-nCoV): a study of 63 patients in Wuhan, China. |
| 41 | Shi et al. 2020 | Clinical characteristics of 101 non-surviving hospitalized patients with COVID-19: A single center, retrospective study. |
| 42 | Li et al. 2020 | Clinical characteristics of 25 death cases infected with COVID-19 pneumonia: a retrospective review of medical records in a single medical center, Wuhan, China. |
| 43 | Zhang et al. 2020 | Clinical characteristics of 82 death cases with COVID-19. |

**Appendix 4 Supplement reference**

W1 World Health Organization. Rolling updates on coronavirus disease (COVID-19) [Updated 23rd April 2020]. Available at: [https://www.who.int/emergencies/diseases/novel-coronavirus-2019/events-as-they-happen](https://www.who.int/emergencies/diseases/novel-coronavirus-2019/events-as-they-happen). Accessed 23 April 2020.

W2 Higgins JPT, Thomas J, Chandler J, Cumpston M, Li T, Page MJ, Welch VA (editors). Cochrane Handbook for Systematic Reviews of Interventions version 6.0 (updated July 2019). Cochrane, 2019. Available from www.training.cochrane.org/handbook.

W3 World Health Organization. Report of the WHO-China Joint Mission on Coronavirus Disease 2019 (COVID-19). February 28, 2020. Available at: [https://www.who.int/docs/default-source/coronaviruse/who-china-joint-mission-on-covid-19-final-report.pdf](https://www.who.int/docs/default-source/coronaviruse/who-china-joint-mission-on-covid-19-final-report.pdf). Accessed 20 March 2020.

W4 CDC COVID-19 Response Team. Severe Outcomes Among Patients with Coronavirus Disease 2019 (COVID-19) —United States, February 12–March 16, 2020. Marth 18, 2020. Available at: https://www.cdc.gov/mmwr/volumes/69/wr/pdfs/mm6912e2-H.pdf. Accessed 20 March 2020.

W5 (Italy) C-SG. Characteristics of COVID-19 patients dying in Italy: Report based on available data on March 26th, 2020. Available at: [https://www.epicentro.iss.it/coronavirus/bollettino/Report-COVID-2019_26_marzo_eng.pdf](https://www.epicentro.iss.it/coronavirus/bollettino/Report-COVID-2019_26_marzo_eng.pdf). Accessed 31 March 2020.

W6 China NHCotPsRo. Novel coronavirus pneumonia prevention and control program (Sixth Edition). Available at: [http://www.nhc.gov.cn/jkj/s3577/202003/4856d5b0458141fa9f376853224d41d7/files/4132bf035bc242478a6eaf157eb0d979.pdf](http://www.nhc.gov.cn/jkj/s3577/202003/4856d5b0458141fa9f376853224d41d7/files/4132bf035bc242478a6eaf157eb0d979.pdf). Accessed 15 March 2020.

W7 Rijksinstituut voor Volksgezondheid en Milieu (RIVM). COVID-19: Nieuwe aanwijzing voor inwoners Noord-Brabant. [COVID-19: Advice for residents of Noord-Brabant]. Bilthoven: RIVM; 2020. Dutch. Available at: [https://www.rivm.nl/nieuws/covid-19-nieuwe-aanwijzing-voor-inwoners-noord-brabant](https://www.rivm.nl/nieuws/covid-19-nieuwe-aanwijzing-voor-inwoners-noord-brabant). Accessed 15 March 2020.

W8 Reusken CB, Buiting A, Bleeker-Rovers C, Diederen B, Hooiveld M, Friesema I, Koopmans M, Kortbeek T, Lutgens SP, Meijer A, Murk JL. Rapid assessment of regional SARS-CoV-2 community transmission through a convenience sample of healthcare workers, the Netherlands, March 2020. Eurosurveillance. 2020 Mar 26;25(12):2000334.

W9 CBC News. Community transmission of COVID-19 detected in Winnipeg, public health officials say. Available from: [https://www.cbc.ca/news/canada/manitoba/manitoba-covid-19-briefing-1.5517379](https://www.cbc.ca/news/canada/manitoba/manitoba-covid-19-briefing-1.5517379). Accessed 5 April 2020.

W10 Localnews8. Community transmission of COVID-19 identified in Teton County, more cases in Custer and Bonneville. Available from: Counties[https://localnews8.com/health/coronavirus/2020/04/01/community-transmission-of-covid-19-identified-in-teton-county-more-cases-in-custer-and-bonneville-counties/](https://localnews8.com/health/coronavirus/2020/04/01/community-transmission-of-covid-19-identified-in-teton-county-more-cases-in-custer-and-bonneville-counties/). Accessed 5 April 2020.

W11 Al Jazeera. Thousands of medical staff infected with coronavirus in Italy. Available at: 

[https://www.aljazeera.com/news/2020/03/rising-number-medical-staff-infected-coronavirus-italy-200318183939314.html](https://www.aljazeera.com/news/2020/03/rising-number-medical-staff-infected-coronavirus-italy-200318183939314.html). Accessed 5 April 2020.

W12 NBC News. Medical workers in Spain and Italy 'overloaded' as more of them catch coronavirus. Available at: [https://www.nbcnews.com/news/world/medical-workers-spain-italy-overloaded-more-them-catch-coronavirus-n1170721](https://www.nbcnews.com/news/world/medical-workers-spain-italy-overloaded-more-them-catch-coronavirus-n1170721). Accessed 5 April 2020.

W13 Nacoti M, Ciocca A, Giupponi A, et al. At the Epicenter of the Covid-19 Pandemic and Humanitarian Crises in Italy: Changing Perspectives on Preparation and Mitigation. NEJM Catalyst Innovations in Care Delivery 1.2 (2020). 

W14 S Spina, F Marrazzo, M Migliari, R Stucchi, A Sforza, R Fumagalli. The response of Milan’s Emergency Medical System to the COVID-19 outbreak in Italy.Lancet. 2020; 395:e49-e50. 

W15 The New York times. Nurses Die, Doctors Fall Sick and Panic Rises on Virus Front Lines. Available at: [https://www.nytimes.com/2020/03/30/nyregion/ny-coronavirus-doctors-sick.html](https://www.nytimes.com/2020/03/30/nyregion/ny-coronavirus-doctors-sick.html). Accessed 5 April 2020.

W16 The Arab Weekly. Poor working conditions hamper Egypt’s medical professionals’ effort to stop spread of virus. Available at: [https://thearabweekly.com/poor-working-conditions-hamper-egypts-medical-professionals-effort-stop-spread-virus](https://thearabweekly.com/poor-working-conditions-hamper-egypts-medical-professionals-effort-stop-spread-virus). Accessed 5 April 2020.

W17 European Centre for Disease Prevention and Control. Infection prevention and control in the household management of people with suspected or confirmed coronavirus disease (COVID-19). Stockholm: ECDC; 2020.

W18 Liu X, Wang L, ALI ST, Du Z, Bosetti P, Cowling BJ, Wu Y. Household transmissions of SARS-CoV-2 in the time of unprecedented travel lockdown in China. MedRxiv. [Preprint]. March 30, 2020. [cited 2020 Mar 31 ]. Available from: [https://www.medrxiv.org/content/10.1101/2020.03.02.20029868v2](https://www.medrxiv.org/content/10.1101/2020.03.02.20029868v2)

W19 Nishiura H, Oshitani H, Kobayashi T, Saito T, Sunagawa T, Matsui T, Wakita T, COVID M, Suzuki M. Closed environments facilitate secondary transmission of coronavirus disease 2019 (COVID-19). MedRxiv. [Preprint].  March 3,2020. [cited 2020 Mar 31 ]. Available from:  [https://www.medrxiv.org/content/10.1101/2020.02.28.20029272v1](https://www.medrxiv.org/content/10.1101/2020.02.28.20029272v1)

W20 British Politics and Policy at LSE. COVID-19: Time to reduce the prison population in England and Wales. Available at: [https://blogs.lse.ac.uk/politicsandpolicy/covid-19-prison-population/](https://blogs.lse.ac.uk/politicsandpolicy/covid-19-prison-population/). Accessed 5 April 2020.

W21 CMU News. Prisoners, Police and the Pandemic: Heinz College criminologist and public policy expert Daniel Nagin discusses disease transmission within the prison system. Available at:  [https://www.cmu.edu/news/stories/archives/2020/march/prisoners-pandemics.html](https://www.cmu.edu/news/stories/archives/2020/march/prisoners-pandemics.html). Accessed 5 April 2020.

W22 ABC News. COVID-19, Cruise ship "nightmare". Available from: [https://www.abc.net.au/chinese/2020-04-04/the-cruises-hit-by-coronavirus/12120864](https://www.abc.net.au/chinese/2020-04-04/the-cruises-hit-by-coronavirus/12120864). Accessed 5 April 2020.

W23 Linton NM, Kobayashi T, Yang Y, Hayashi K, Akhmetzhanov AR, Jung SM, et al. Incubation Period and Other Epidemiological Characteristics of 2019 Novel Coronavirus Infections with Right Truncation: A Statistical Analysis of Publicly Available Case Data. Journal of clinical medicine. 2020 Feb 17;9(2). 538.

W24 Backer JA, Klinkenberg D, Wallinga J. Incubation period of 2019 novel coronavirus (2019-nCoV) infections among travellers from Wuhan, China, 20-28 January 2020. Eurosurveillance 25.5 (2020).Feb;25(5).

W25 Song Qianqian, Zhao Han, Fang Liqun, Liu Wei, Zheng Chuang, Zhang Yong. Study on assessing early epidemiological parameters of coronavirus disease epidemic in China. Chinese Journal of Epidemiology. 2020, 41(4): 461-465. 

W26 Wang P, Lu J, Jin Y, Zhu M, Wang L, Chen SJm. Epidemiological characteristics of 1212 COVID-19 patients in Henan, China. MedRxiv. [preprint] Feburary 25, 2020. [cited 2020 Mar 20]. Available from: [https://www.medrxiv.org/content/10.1101/2020.02.21.20026112v2.full.pdf](https://www.medrxiv.org/content/10.1101/2020.02.21.20026112v2.full.pdf)

W27 Li J, Wang Y, Gilmour S, Wang M, Yoneoka D, Wang Y, et al. Estimation of the epidemic properties of the 2019 novel coronavirus: A mathematical modeling study. 2020. Available at SSRN: [https://ssrn.com/abstract=3542150](https://ssrn.com/abstract=3542150). Accessed 5 April 2020. 

W28 Wu JT, Leung K, Leung GM. Nowcasting and forecasting the potential domestic and international spread of the 2019-nCoV outbreak originating in Wuhan, China: a modelling study. The Lancet. 2020 Jan 31.

W29 Shen M, Peng Z, Xiao Y, Zhang LJb. Modelling the epidemic trend of the 2019 novel coronavirus outbreak in China. BioRxiv. [preprint]. January 25, 2020. [cited 2020 Mar 20]. Available from:  [https://www.biorxiv.org/content/10.1101/2020.01.23.916726v1.full.pdf](https://www.biorxiv.org/content/10.1101/2020.01.23.916726v1.full.pdf)

W30 Zhao S, Lin Q, Ran J, Musa SS, Yang G, Wang W, et al. Preliminary estimation of the basic reproduction number of novel coronavirus (2019-nCoV) in China, from 2019 to 2020: A data-driven analysis in the early phase of the outbreak. International journal of infectious diseases : IJID : official publication of the International Society for Infectious Diseases. 2020 Jan 30;92:214-7.

W31 Read JM, Bridgen JR, Cummings DA, Ho A, Jewell CPJm. Novel coronavirus 2019-nCoV: early estimation of epidemiological parameters and epidemic predictions. MedRxiv. [preprint]. January 28, 2020. [cited 2020 Mar 20]. Available from:  [https://www.medrxiv.org/content/10.1101/2020.01.23.20018549v2.full.pdf](https://www.medrxiv.org/content/10.1101/2020.01.23.20018549v2.full.pdf)

W32 Zhang S, Diao M, Yu W, Pei L, Lin Z, Chen DJIJoID. Estimation of the reproductive number of Novel Coronavirus (COVID-19) and the probable outbreak size on the Diamond Princess cruise ship: A data-driven analysis. International Journal of Infectious Diseases (2020).

W33 Liu T, Hu J, Xiao J, He G, Kang M, Rong Z, et al. Time-varying transmission dynamics of Novel Coronavirus Pneumonia in China. BioRxiv. [preprint]. Feburary 13, 2020. [cited 2020 Mar 20]. Available from:  [https://www.biorxiv.org/content/10.1101/2020.01.25.919787v2.full.pdf](https://www.biorxiv.org/content/10.1101/2020.01.25.919787v2.full.pdf)

W34 Tang B, Wang X, Li Q, Bragazzi NL, Tang S, Xiao Y, et al. Estimation of the transmission risk of the 2019-nCoV and its implication for public health interventions. Journal of Clinical Medicine 9.2 (2020): 462. 

W35 Liu T, Hu J, Kang M, Lin L, Zhong H, Xiao J, et al. Transmission dynamics of 2019 novel coronavirus (2019-nCoV). Available at SSRN: [https://ssrn.com/abstract=3526307](https://ssrn.com/abstract=3526307). Accessed 5 April 2020.

W36 Bauch CT, Lloyd-Smith JO, Coffee MP, Galvani APJE. Dynamically modeling SARS and other newly emerging respiratory illnesses: past, present, and future. 2005: 791-801.

W37 Bauch CT, Oraby TJTL. Assessing the pandemic potential of MERS-CoV. 2013; 382(9893): 662-4.

W38 European Centre for Disease Prevention and Control. Download today’s data on geographic distribution of COVID-19 cases worldwide; 2020 [Cited 22 April 2020]. Available from: 

[https://www.ecdc.europa.eu/en/publications-data/download-todays-data-geographic-distribution-covid-19-cases-worldwide](https://www.ecdc.europa.eu/en/publications-data/download-todays-data-geographic-distribution-covid-19-cases-worldwide)

W39 CCTV News. The diagnosis of 30 hour old infants in Wuhan may have mother infant vertical infection. 2020 Feb 5. Available from:[http://news.cctv.com/2020/02/05/ARTIBNDLKFslreOGo0FW6Geu200205.shtml](http://news.cctv.com/2020/02/05/ARTIBNDLKFslreOGo0FW6Geu200205.shtml). Accessed 20 March 2020.

W40 World Health Organization. Update 30 - Status of diagnostic test, significance of “super spreaders”, situation in China. 2003 Apr 15. Available from: https://www.who.int/csr/sars/archive/2003_04_15/en/. Accessed 21 March 2020.

W41 The Paper. COVID-19 "Fu Yang": there is no unified conclusion, and discharge management is being strengthened. 2020 Mar 5. Available from:[https://www.thepaper.cn/newsDetail_forward_6341027](https://www.thepaper.cn/newsDetail_forward_6341027). Accessed 21 March 2020.

W42 The Paper. Zhong Nanshan: at present, it seems that Fuyang patients are not infectious. 2020 Mar 12. Available from: [https://www.thepaper.cn/newsDetail_forward_6473045](https://www.thepaper.cn/newsDetail_forward_6473045). Accessed 21 March 2020.

W43 Euronnews. Coronavirus: What is Kawasaki disease and its possible link with COVID-19 in children? 2020 Apr 5. Available from:

[https://www.euronews.com/2020/05/04/coronavirus-what-is-kawasaki-disease-and-its-possible-link-with-covid-19-in-children](https://www.euronews.com/2020/05/04/coronavirus-what-is-kawasaki-disease-and-its-possible-link-with-covid-19-in-children). Accessed 20 April 2020.
